# Uniportal surgical biopsy, without orotraqueal intubation, without thoracic drainage in intersticial pulmonary disease: initial results

**DOI:** 10.1590/0100-6991e-20202914

**Published:** 2021-05-31

**Authors:** JULIANO MENDES SOUZA, IGHOR RAMON PALLU DORO PEREIRA, ARIELA VICTÓRIA BORGMANN, RAFAEL ENRIQUE CHIARADIA, PAULO CESAR BUFFARA BOSCARDIM

**Affiliations:** 1 - Faculdades Pequeno Príncipe, Curso de Medicina - Curitiba - PR - Brasil; 2 - Hospital Nossa Senhora das Graças, Departamento de Cirurgia Torácica - Curitiba - PR - Brasil

**Keywords:** Thoracic Surgery, Pneumopathies, Image-Guided Biopsy, Thoracic Surgery, Video-Assisted, Cirurgia Torácica, Pneumopatias, Biópsia Guiada por Imagem, Cirurgia Torácica Vídeoassistida

## Abstract

**Objective:**

interstitial lung disease comprises a group of lung diseases with wide pathophysiological varieties. This paper aims to report the video thoracoscopic surgical biopsy in patients with interstitial lung disease through a single minimal chest incision, without orotracheal intubation, without chest drainage, and without the use of neuromuscular blockers.

**Methods:**

this study is a series of 14 cases evaluated retrospectively, descriptively, where patients underwent a pulmonary surgical biopsy from January 2019 to January 2020. The patients included in the study had diffuse interstitial lung disease without a defined etiological diagnosis.

**Results:**

none of the patients had transoperative complications, there was no need for chest drainage in the postoperative period, and the patients pain, assessed using the verbal scale, had a mode of 2 (minimum value of 1 and maximum of 4) in the post immediate surgery and 1 (minimum value of 1 and maximum of 3) at the time of hospital discharge. The length of hospital stay was up to 24 hours, with 12 patients being discharged on the same day of hospitalization.

**Conclusion:**

therefore, it is concluded in this series of cases that the performance of uniportal video-assisted thoracoscopic surgery procedures to perform lung biopsies, without orotracheal intubation, without chest drainage, and without the use of neuromuscular blockers, bring benefits to the patient without compromising his safety. Further larger studies are necessary to confirm the safety and efficiency of this method.

## RESUMO

**Objetivo:**

a doença pulmonar intersticial compreende um grupo de doenças pulmonares com grandes variedades fisiopatológicas. Este trabalho objetiva relatar a biópsia cirúrgica videotoracoscópica em pacientes com doença pulmonar intersticial por meio de incisão torácica mínima única, sem intubação orotraqueal, sem drenagem torácica e sem uso de bloqueadores neuromusculares.

**Métodos:**

este estudo é uma série de 14 casos avaliados de forma retrospectiva, descritiva, onde no qual os pacientes foram submetidos a biópsia cirúrgica pulmonar no período de janeiro de 2019 a janeiro de 2020. Os pacientes incluídos na pesquisa, apresentavam doença pulmonar intersticial difusa sem diagnóstico etiológico definido.

**Resultados:**

nenhum dos pacientes apresentou complicações transoperatórias, não houve necessidade de drenagem torácica no período pós-operatório e a dor dos pacientes, avaliada por meio da escala verbal, teve moda de 2 (valores mínimos de 1 e máximo de 4) no período de pós-operatório imediato e 1 (valores mínimos de 1 e máximos de 3) no momento da alta hospitalar. O tempo de permanência hospitalar foi de até 24 horas, sendo que 12 pacientes receberam alta no mesmo dia da internação.

**Conclusão:**

conclui-se, assim, que nesta série de casos, a realização de procedimentos de cirurgia toracoscópica videoassistida uniportais para realização de biópsias pulmonares, sem intubação orotraqueal, sem drenagem torácica e sem uso de bloqueadores neuromusculares trazem benefícios para o paciente sem comprometer sua segurança. Estudos maiores são necessários para comprovar tanto a segurança quanto à eficácia deste método.

## INTRODUCTION

Interstitial lung disease (ILD) comprises a group of lung conditions with a wide range of etiologies, pathology, treatments, and prognosis^1^. Patients with ILD often have no confirmed etiologic diagnosis and appropriate treatment instituted because they cannot be subjected to the risk of standard surgical treatment. However, with the development of minimally invasive surgical techniques and advances in anesthesia, patients at higher surgical risk, as well as those with higher risk of exacerbation of the underlying lung disease, can undergo a thoracoscopic procedure^2^ ^,^ ^3^.

Video-assisted thoracoscopic surgery (VATS) is now a well-established technique for lung biopsy. It is safe, allows smaller incisions, shorter hospital stay, less postoperative pain, bleeding, and damage to the pulmonary function, and causes minimal postoperative discomfort^1^ ^,^ ^2^. Traditionally, routine placement of an intercostal chest tube has been an established part of VATS lung biopsy, as has intubation with double-lumen tubes^3^. However, complications associated with intubation, including pulmonary infections, lung injury due to pressure from ventilation or overexpansion, arrhythmia and cardiac dysfunction, bronchospasm, postoperative sore throat, and irritative cough still pose concerns^3^.

To reduce the complications related to conventional anesthesia with tracheal intubation during thoracic surgery, sedation with epidural anesthesia has been used for various VATS procedures^4^, although general anesthesia and monopulmonary mechanical ventilation is considered the standard of care in this scenario^3^. In addition, spontaneous ventilation anesthesia (SVA) and uniportal approaches have become notable evolutionary stages in VATS. Strategies to reduce trauma related to the operation are reduction of postoperative pain and wound paresthesia, no residual neuromuscular block, and no orotracheal intubation, which is related to airway trauma, postoperative cough, and other deleterious effects^5^. Also, the early removal of the intercostal chest tube after VATS lung biopsy reduces pain without increasing postoperative complications^2^.

This study aims to report a case series of thoracoscopic surgical biopsies in patients with interstitial lung disease through a single minimum thoracic incision without intubation or chest drainage, and without the use of neuromuscular blockers.

## METHODS

This study is a series of cases evaluated retrospectively and descriptively, in which patients underwent pulmonary surgical biopsy from January 2019 to January 2020. This work was approved by the Human Research Ethics Committee under number: 39836220.6.0000.5580. The included patients had diffuse interstitial lung disease without a defined etiological diagnosis. In these cases, the definitive diagnosis and treatment could not be performed after tomographic, laboratory, or radiological evaluations. Patients with disease in advanced stages were not included, according to the Guidelines for Interstitial Lung Diseases of the Brazilian Society of Pulmonology and Tisiology^6^. Criobiopsy was not performed on any of the patients involved due to unavailability of this procedure in the research institution during the evaluation period.

After monitoring the patient, we performed the intravenous infusion of propofol to anesthesia induction, followed by remifentanil to maintain anesthesia and assist in controlling the respiratory rate during the procedure. Then, we inserted a laryngeal mask for airway maintenance. No neuromuscular blockers were used during the procedures, allowing the patient to maintain spontaneous ventilation or in synchronized intermittent mandatory mode (SIMV).

**Figure 1:**
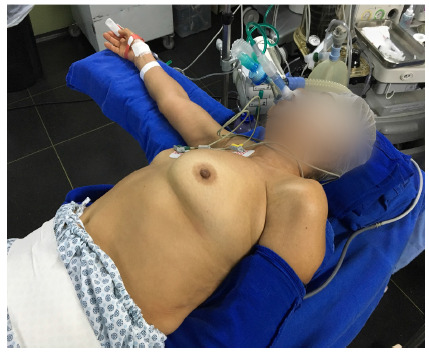
Position of the patient in the oblique position, with placement of a lateral cushion and homolateral upper limb close to the body..

**Figure 2:**
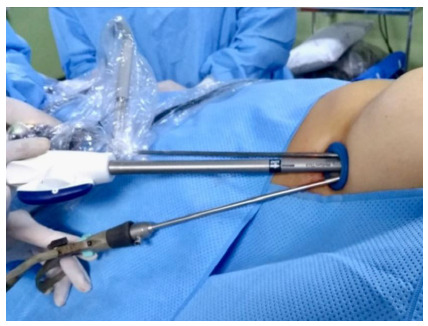
Positioning of the surgical instruments: Snowden-Pencer articulated grasper, 5mm opticque and a 30º endoscopic stapler with a purple 60mm charge.

After positioning the patient (Figure 1), we performed anesthesia at the incision site and preemptive intercostal block with a solution of bupivacaine, dexamethasone, and clonidine. We performed a single 2.5 cm long incision in the 4th or 5th intercostal space between the mid and the anterior axillary lines, to execute the uniportal technique (Figure 2).

The definition of the biopsy site was followed the Computerized Tomography exam from the preoperative period. In all procedures, we removed two fragments from distinct lung lobes for anatomopathological analysis by wedge resection and endoscopic stapling. After the procedure, the patient’s lung expansion was performed, using suction and positive pressure ventilation. Then, we conducted the sealed underwater test to check for the presence of air leakage. For this test, we inserted a 14F nasogastric catheter through the uniportal incision, with its tip positioned anteriorly and at the apex of the pleural cavity. The other end remained immersed in saline solution, at a level below the thoracic cavity, acting as a water seal drain. Then we manually and gradually inflated the lungs with positive pressure until reaching 30 cm/H_2_O, while tending to the ventilator monitor. After each inflation, we made a sustained inspiratory pause of 10 seconds, to observe any air escape in the water seal. The absence of air leak in this test ruled out the need for a chest tube in the postoperative period.

For the assessment of lung function, as these patients have disease with many variations and rapid progression, including exacerbations, we opted for spirometry on the day of hospitalization, with a portable equipment in the operating room. As a service routine, we used the same equipment in the postoperative evaluation, seeking more reliability. We carried out a new evaluation 24 hours after the procedure, the critical moment of decreased pulmonary function related to the surgical procedure itself, ensuring the absence of any anesthetic medication effect and with effective pain control^1^ ^-^ ^3^.

## RESULTS

From January 2019 to January 2020, 14 patients with interstitial lung disease underwent diagnostic pulmonary wedge resection in two lobes with an endoscopic stapler, through a 2.5 cm uniportal surgical access, under general venous anesthesia without the use of muscle relaxants, ventilatory control by laryngeal mask, and without the use of a chest tube in the postoperative period.

 There was an equal distribution between women and men, with an average age of 65.8 years (ranging between 37.0 and 81.0). The evolution data of the patients are listed in Table 1. We assessed the pulmonary function immediately before anesthetic induction, by forced expiratory volume in one second (FEV1), with mean values of 2.04 liters (range 1.40-2.76), corresponding to 65.9% (varying between 54.1% and 97.6%) of the predicted value.

**Table 1:** Surgical results.

Variable	Values	
Women	7	50%
Age (years)	65.8^1^	(37.0-81.0)^2^
FEV1 pre (liters)	2.0^1^	(1.4-2.7)^2^
FEV1 post (IPO - liters)	1.8^1^	(1.2-2.5)^2^
FEV1 pre (predicted percentage)	65.9^1^	(54.1-97.6)^2^
FEV1 post (IPO - predicted percentage)	58.7^1^	(44.9-91.4)^2^
Surgery time (minutes)	57.7^1^	(39.0-78.0)^2^
Pain score in the IPO	2^3^	(1-4)^2^
Pain scale at discharge	1^3^	(1-3)^2^
Need for SIMV	8	57.1%
Transoperative complications	0	0%
Need for chest drainage	0	0%
Conversion to open surgery	0	0%
Residual Pneumothorax 5% (up to 2 cm)	2	14.2%
Postoperative complications	2	14.2%
Definitive diagnosis	14	100.0 %

^1^Averages; ^2^Minimum and maximum values; ^3^Mode; IPO-Immediate post operative period.

 None of the patients had transoperative complications, and it was possible to maintain spontaneous ventilation by laryngeal mask without the need to convert the procedure to multiportal VATS, open thoracotomy, or endotracheal intubation. There was no need for chest drainage in the postoperative period in any of the patients. In two patients (14.20%), we identified a minimal residual pneumothorax (less than 2 cm) by chest radiograph in the apical region, which did not require intervention. One patient had a subclinical atelectasis (7.14%), which improved with respiratory physiotherapy during hospitalization. In one patient, pulmonary thromboembolism was identified in the late postoperative period, without the need for a new hospitalization.

We assessed patients’ pain by means of a verbal scale, ranging from 1 (almost nonexistent pain) to 10 (unbearable pain). In the immediate postoperative period, the mode of verbal pain scale values was 2 (range 1-4). We used the same scale to assess pain at discharge, the mode of values being 1 (range 1-3). The length of hospital stay was up to 24 hours, with 12 patients being discharged on the same day of hospitalization. The average lung capacity at discharge as measured by the FEV1 was 1.83 liters (range 1.21-2.55), equivalent to 58.7% (range 44.9%-91.4%) of the predicted value.

 We maintained spontaneous ventilation for all patients. During lung stapling, however, it was necessary to titrate the remifentanil to decrease the diaphragm movements, which required 57.1% of patients to be kept on SIMV ventilation during this period.

**Figure 3:**
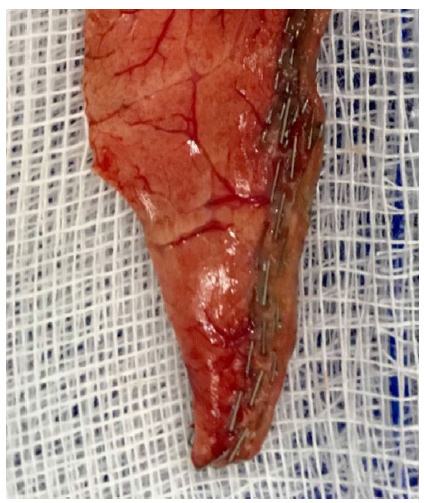
Sample of pulmonary tissue.

 The samples taken for biopsy were sufficient to obtain the diagnosis in all patients (Figure 3).

## DISCUSSION

 Interstitial lung diseases are characterized by a process of thickening of the alveolar septa, followed by fibroblast proliferation with collagen deposition, which can progress to pulmonary fibrosis when not controlled. More than 200 subtypes of ILD have been reported, making the diagnosis of these patients a great challenge when based only on clinical history and on laboratory and imaging exams^7^. It has been reported that only 15% of patients with ILD had an accurate diagnosis without performing a Surgical Lung Biopsy (SLB)^8^. Computed tomography (CT) has diagnostic accuracy between 61% and 80% of cases, as well as bronchoalveolar lavage tests and transbronchial lung biopsy. However, these have limited diagnostic utility and efficacy, causing approximately one third of patients to need SLB for definite diagnosis^7^.

 Uniportal VATS procedures for lung biopsies and for other thoracic procedures has increased in recent years, with demonstrated benefits to the patient without compromising safety^9^. A study with 1,063 patients reported a zero rate of intraoperative or 30-day mortality after uniportal surgical procedures^10^ ^,^ ^11^, the same as in the present case series.

Thus, procedures without endotracheal intubation have been proven safe. Although conversion to open surgery has been described in 4.4% of cases, 95% of them had no transoperative complications^5^. In this study, there was no need for conversion to endotracheal intubation in any patient and there were no transoperative complications. There are reports of postoperative complications between 6.9 and 7.1% of patients^1^ ^,^ ^5^ ^,^ ^12^, lower than our result, which was 14.2%. This can be attributed to the small number. This observation would need to be confirmed in a larger sample.

 The performance of uniportal VATS in patients with ILD under general anesthesia, with endotracheal intubation and positive pressure pulmonary ventilation (PPV) is common. However, mortality rates in these cases vary between 1.5 and 3.6%, with morbidity rates of 16% after the procedure^12^. These values may be strongly associated with the triggering of adverse events from intubation and mechanical ventilation with positive pressure, such as the triggering of alveolar barotrauma, volutrauma, and atelectasis, which increase the risk of pneumonia^13^.

The residual neuromuscular block of the procedure can lead the patient to depend on mechanical ventilation in the postoperative period, increasing the risks of sepsis and neuromyopathy^13^. When on spontaneous ventilation, pulmonary and alveolar collapse is never complete, unlike the procedure with endotracheal intubation. In these cases, the absence of complete pulmonary collapse favors the patient with ILD^13^. A study comparing bronchoalveolar lavage findings before and after VATS in patients undergoing procedures with endotracheal intubation and with spontaneous ventilation found that in the latter group the level of inflammatory cytokines in the postoperative period was considerably lower^5^.

Another great advantage of VATS procedures for the treatment of chest diseases is the feasibility and safety of not using chest drains in the immediate postoperative period. The use of endoscopic staplers and the meticulous suture technique prevent air leak after the procedure, which justify not performing drainage^4^. However, the use of the endoscopic staplers is still limited in Brazil, due to the unavailability in most of the services of the Unified Health System (SUS), though being routine in the supplementary health system. In this series, no patient required chest drainage in the postoperative period and the two patients who presented residual pneumothorax (apical, up to 2 cm), had no clinical repercussions, requiring no intervention. This residual pneumothorax index is close to 15%, according to another study^4^. Furthermore, no other complications were associated with not using the drain in the patients involved in this series.

VATS alone causes little discomfort in the postoperative period^4^ ^,^ ^14^. Studies have shown that patients who used a chest tube after a surgical procedure reported pain even after tube removal, while patients who had a tube reported less severe pain, and for a shorter period^4^. In Germany, a randomized study used a numerical pain scale to compare the level of discomfort of patients who underwent chest tube placement after surgery and those who did not. In 24 hours, the group without the drain reported a decrease in pain from levels close to 4 to values below 2. On the other hand, in the group with a chest drain, the values remained close to 6, without significant reduction^15^. In our series, which also used the numerical pain scale in the immediate postoperative period, the mode of pain scale scores were 2 (ranging from 1 to 4), and at discharge, 1 (ranging from 1 to 3), showing the positive contribution to reducing patient discomfort when using this protocol. These values have a significant impact on patients and on the health system, since many chronic opioid analgesic users have them initiated for the treatment of acute pain after surgical procedures, being subject to their adverse effects, including the need of prolonged use and development of chronic pain^16^.

Surgical techniques with a minimally invasive approach, anesthetic procedures that allow the patient to remain on spontaneous ventilation, and the non-use of chest drains after the operation directly contribute to the shortening of the patient’s hospital stay. Each of these protocols allows the patient to recover faster after the procedure^17^. Several are the described forms of diaphragm movement interruption for the completion of surgery with contralateral spontaneous ventilation, the anesthetic phrenic nerve block being the most widespread^17^. For patients with ILD, this block would be harmful, since the time elapse before the diaphragm movement return depends on the anesthetic metabolism. This blockage favors the appearance of atelectasis and hypoventilation. We decided to keep the patient on spontaneous ventilation without blocking the diaphragm to prevent this complication. In 57.1% of patients during the lung parenchyma stapling, it was necessary to reduce the diaphragm movements, achieved with increasing the remifentanil infusion. Being a high potency and fast metabolism opioid, it enabled the time needed for stapling, the patient remaining on SIMV ventilation during this period.

In this study, the discharge was early, and the maximum length of stay for all patients was 24 hours, 12 being discharged at the same day of admission.

 All patients in this study had a definitive diagnosis through biopsy performed via uniportal VATS, corroborating the results of 95% yield (ranging from 85% to 100%) from other studies^8^ ^,^ ^18^.

Despite limitations due to sample size and design, this series demonstrates that it is possible to perform this type of less invasive procedure in this severe disease that often lacks a definitive anatomopathological diagnosis and adequate treatment. Undoubtedly, further studies are needed to prove both the safety and the effectiveness of this method.

## References

[1] Peng G, Liu M, Luo Q, Chen H, Yin W, Wang W (2017). Spontaneous ventilation anesthesia combined with uniportal and tubeless thoracoscopic lung biopsy in selected patients with interstitial lung diseases. J Thorac Dis.

[2] Satherley LK, Luckraz H, Rammohan KS, Phillips M, Kulatilake NEP, O'Keefe PA (2009). Routine placement of an intercostal chest drain during video-assisted thoracoscopic surgical lung biopsy unnecessarily prolongs in-hospital length of stay in selected patients. Eur J Cardiothoracic Surg.

[3] Liu J, Cui F, Pompeo E, Gonzalez-Rivas D, Chen H, Yin W (2016). The impact of non-intubated versus intubated anaesthesia on early outcomes of video-assisted thoracoscopic anatomical resection in non-small-cell lung cancer A propensity score matching analysis. Eur J Cardiothoracic Surg.

[4] Luckraz H, Rammohan KS, Phillips M, Abel R, Karthikeyan S, Kulatilake NEP (2007). Is an Intercostal Chest Drain Necessary After Video-Assisted Thoracoscopic (VATS) Lung Biopsy. Ann Thorac Surg.

[5] Liu J, Cui F, Li S, Chen H, Shao W, Liang L (2015). Nonintubated video-assisted thoracoscopic surgery under epidural anesthesia compared with conventional anesthetic option A randomized control study. Surg Innov.

[6] Baldi BG, Pereira CAC (2012). Diretrizes de Doenças Pulmonares Intersticiais da Sociedade Brasileira de Pneumologia e Tisiologia. J Bras Pneumol.

[7] Morris D, Zamvar V (2014). The efficacy of video-assisted thoracoscopic surgery lung biopsies in patients with interstitial lung disease A retrospective study of 66 patients. J Cardiothorac Surg.

[8] Kayatta MO, Ahmed S, Hammel JA, Fernandez F, Pickens A, Miller D (2013). Surgical Biopsy of Suspected Interstitial Lung Disease Is Superior to Radiographic Diagnosis. Ann Thorac Surg.

[9] Fernandez-Pineda I, Seims AD, Vanhouwelingen L, Abdelhafeez H, Wu H, Wu J (2019). Modified Uniportal Video-Assisted Thoracic Surgery Versus Three-Port Approach for Lung Nodule Biopsy in Pediatric Cancer Patients. J Laparoendosc Adv Surg Tech.

[10] Xie D, Wang H, Fei K, Chen C, Zhao D, Zhou X (2016). Single-port video-assisted thoracic surgery in 1063 cases A single-institution experience. Eur J Cardiothoracic Surg.

[11] Gonzalez-Rivas D, Paradela M, Fernandez R, Delgado M, Fieira E, Mendez L (2013). Uniportal video-assisted thoracoscopic lobectomy Two years of experience. Ann Thorac Surg.

[12] Pompeo E, Rogliani P, Atinkaya C, Guerrera F, Ruffini E, Iñiguez-Garcia MA, Peer M, Voltolini L, Caviezel C, Weder W, Opitz I, Cavalli F, Sorge R, ESTS awake thoracic surgery working group (2019). Nonintubated surgical biopsy of undetermined interstitial lung disease A multicentre outcome analysis. Interact Cardiovasc Thorac Surg.

[13] Kiss G, Castillo M (2015). Nonintubated anesthesia in thoracic surgery General issues. Ann Transl Med.

[14] Ayed AK, Raghunathan R (2000). Thoracoscopy versus open lung biopsy in the diagnosis of interstitial lung disease a randomised controlled trial. J R Coll Surg Edinb.

[15] Lesser T, Doenst T, Lehmann T, Mukdessi J (2019). Lung biopsy without pleural drainage A randomized study of a commonly performed video-thoracoscopic procedure. Dtsch Arztebl Int.

[16] Brat GA, Agniel D, Beam A, Yorkgitis B, Bicket M, Homer M (2018). Postsurgical prescriptions for opioid naive patients and association with overdose and misuse: retrospective cohort study. BMJ.

[17] Batchelor TJP, Rasburn NJ, Abdelnour-Berchtold E, Brunelli A, Cerfolio RJ, Gonzalez M (2019). Guidelines for enhanced recovery after lung surgery Recommendations of the Enhanced Recovery after Surgery (ERAS(r)) Society and the European Society of Thoracic Surgeons (ESTS). Eur J Cardiothoracic Surg.

[18] Kim TH, Cho JH (2020). Nonintubated Video-Assisted Thoracoscopic Surgery Lung Biopsy for Interstitial Lung Disease. Thorac Surg Clin.

